# Unmet Supportive Care Needs of Survival Patients with Nasopharyngeal Carcinoma

**DOI:** 10.3390/ijerph17103519

**Published:** 2020-05-18

**Authors:** Ya-Ling Lin, Chun-Yi Chuang, Vivian Chia-Rong Hsieh, Ming-Shou Tsai, Yen-Fang Liu, Xian-Xiu Chen, Shwn-Huey Shieh

**Affiliations:** 1 Department of Public Health, China Medical University, Taichung 40402, Taiwan; yaling80372@gmail.com; 2 Department of Nursing, Taichung Hospital, Ministry of Health Welfare, Taichung 40343, Taiwan; 3 School of Medicine, Chung Shan Medical University, Taichung 40201, Taiwan; cyi4602@gmail.com; 4 Department of Otolaryngology, Chung Shan Medical University Hospital, Taichung 40201, Taiwan; 5 Department of Health Services Administration, China Medical University, Taichung 40402, Taiwan; crhsieh@mail.cmu.edu.tw; 6 Department of Otorhinolaryngology, China Medical University Hospital, Taichung 40402, Taiwan; minghsui@mail.cmuh.org.tw; 7 Department of Nursing, China Medical University Hospital, Taichung 40402, Taiwan; n4808@mail.cmuh.org.tw; 8 Chinese Medicine Research Center, China Medical University, Taichung 40402, Taiwan; txxchen@gmail.com; 9 Research Center for Chinese Herbal Medicine, China Medical University, Taichung 40402, Taiwan; 10 Department of Nursing, Asia University, Taichung 41354, Taiwan

**Keywords:** unmet care needs, survival patients, nasopharyngeal carcinoma, cancer stages and treatment

## Abstract

This study examined unmet supportive care needs for nasopharyngeal carcinoma (NPC) patients by cancer stage and treatment phase, as well as the factors associated with these unmet needs. At a cancer center in central Taiwan, information on consultations and services patients received at the resource center was described in the service chart. We extracted data available for NPC patients to evaluate their unmet supportive care needs (health information, patient care, treatment, nutritional, psychosocial, and economic) and their association with sex, age, cancer stage, and treatment phase. The 145 NPC patients were 68.3% male, 60.0% less than 50 years old, and 83.5% diagnosed at stages III and IV. The most prevalent unmet need was nutritional (40.7%), followed by psychosocial and patient care, with economic unmet needs the least (4.8%). Women were more likely than men to have patient care unmet needs (32.6% vs. 15.2%). Nutritional unmet need was higher in older patients than in younger ones (83.3% vs. 35.6%), with an adjusted odds ratio (aOR) of 9.39 (95% confidence interval (CI) = 2.17–40.70). Psychosocial unmet needs were higher in younger patients than old patients (34.5% vs. 0%) and in patients interviewed during follow-up period than those at newly diagnosed (55.2% vs. 23.1%). In conclusion, the most commonly reported concern was nutritional unmet needs for NPC patients. Their unmet needs may vary by demographic and disease factors, including patient sex and age, cancer stage, and treatment phase.

## 1. Introduction

Nasopharyngeal cancer (NPC) is a common cancer originating in the nasopharynx and upper throat, with approximately 90,000 patients worldwide diagnosed annually, comprising 0.7% of all cancers [1]. The disease is prevalent in Asian population, and has been associated with the consumption of preserved foods (e.g., salted fish) and nitrite-containing foods, as well as with the Epstein-Barr virus infection [2,3]. This disease accounted for 1.43% of all malignant tumors newly diagnosed in 2016 as the 12th leading cancer in men of Taiwan, with the incidence 2–3 folds greater in males than in females [4]. Patients with NPC need supportive care, as do patients of other types of cancer [5,6,7,8,9,10,11]. Chemotherapy and radiation therapy are often the preferred treatment modality for these patients because NPC tumors occur mainly in the head and neck area, which precludes surgery as a treatment option [12,13]. Patients with NPC receiving chemotherapy and radiation therapy often experience xerostomia and damaged painful oral mucosa. Although the five-year survival rate of NPC has increased to 80% [1,4], survivors may have needs relating to healthcare system and health information, such as patient support, physical and daily living needs, nutrition needs, and sexual, emotional, psychological, and financial needs [14,15,16,17]. The quality of life (QoL) of survivors may be adversely affected during disease progression and treatment, and the survival period [18,19,20,21,22].

Supportive care aims to help patients with their physiological, psychosocial, and spiritual needs across the different disease care phases, from diagnosis to active treatment, relapse, and survivorship [5,23]. The supportive services may restore their emotional stability, improve their social adaptation ability and cognitive functioning, and reduce their physical distress [10,24,25,26].

Nevertheless, much of patients’ supportive care needs remains unmet. In a literature review, Harrison et al. [6] compiled 94 relevant studies to examine the unmet needs for patients with cancer at different cancer stages. The results show that the highest and most unmet needs occurred during the treatment period. Using medical records of 614 Pennsylvanian patients with mixed types of cancer from 1986 to 2005, Barg et al. [27] found that most patients had experienced at least one unmet psychosocial need, especially emotional, physical, and treatment needs, and health education need. Patients of younger age, with more advanced cancer stage and lower income result in more unmet needs [26]. However, Edib et al. [5] reported that more than 65% of Malaysian breast cancer patients were depressed. The unmet needs were particularly prevalent in patients with an older age and early-stage cancer. By contrast, patients with high income who can enjoy high-quality and comprehensive care experienced fewer negative effects of cancer as well as relatively fewer unmet needs [28,29].

Malnutrition is an unmet nutritional need in cancer patients with a great impact in their QoL [30,31,32,33,34]. Patients with advanced stage and older age are more likely exhibit poor QoL with severe malnutrition. Kaiser et al. [31] found that, in 4775 older patients with cancers, 46.2% of patients were malnourished, mainly women. Qiu et al. [32] investigated 159 patients with NPC who experienced significant weight loss after receiving radiation therapy had been suffered from malnutrition, poor QoL associated with advanced cancer stages, insomnia, and fatigue. Patients with a high body mass index before treatment are at an elevated risk of weight loss. Studies found patients with higher educational attainment tend to have fewer unmet nutritional needs [31,34].

In summary, to identify unmet supportive care needs is an essential component of healthcare for cancer patients. Unmet needs appear to relate to the stage of cancer, disease trajectory, treatment phase, and specific diagnosis. Limited studies have investigated the unmet supportive care needs for NPC patients [32]. The purpose of this study was to examine the unmet needs at different cancer stages and treatment phases across six domains (health information, patient care, treatment, nutritional, psychosocial, and economic domains) for NPC patients who had received care at a medical center in central Taiwan. Clinical and socio-demographic factors associated with the unmet needs were evaluated as well.

## 2. Materials and Methods

### 2.1. Study Design and Participants

The medical center is the largest one in Central Taiwan consisting of 8 major departments to serve diverse population with nearly 2100 beds, including a Cancer Center. At the Cancer Center, a “Cancer Resources Center (CRC)” was established to provide consultations and services to cancer patients who received care at the medical center. Patients were encouraged to meet the care team at the resources center, which consisted of an oncology case manager, dietitian, and psychiatrist, at the time of their cancer diagnosis, during hospitalization, and at a follow-up appointment after discharge. During each visit, conversations with patients included discussions of their healthcare needs. The care team completed a narrative record for each visit and then, for patients who had information of unmet need recorded, data on socio-demographic and clinical characteristics, unmet need, and service use were extracted by the research team. For patients with information of unmet need complain available in the records, the patients were selected in our study. For this study, information on patient characteristics, unmet care needs consultations, and services received was extracted from the resource center’s records for eligible patients who received care from the center during 2015–2017. Patients hospitalized for NPC with the following characteristics were considered eligible: (1) pathological biopsy confirmed diagnosis of NPC; (2) aged 20 or older; (3) had received treatment and agreed to participate in this present study; and (4) able to communicate in Mandarin Chinese. In total, 29 patients were excluded because of incomplete data, thus resulting in a final sample of 145 NPC patients eligible to be included in this study. This study was approved by the Research Ethics Committee of China Medical University (CMUH105-REC1-054).

### 2.2. Data Management 

The unmet needs of NPC patients in this study were the data presented by analyzing the unmet needs of patients who had sought assistance from the CRC for issues related to each unmet need domain according to the patients’ chief complaints. Information extracted for this study included demographic status (age and sex), treatment phases, cancer stage, and unmet need items. The unmet needs of NPC patients were divided into six domains, namely health information, patient care, treatment, nutritional, psychosocial, and economic unmet needs. We used “yes or no” to indicate whether the patient had an unmet need for each domain. Health information unmet needs included health education, disease prevention, and explanations of examination procedures. Patient care unmet needs included those related to pain management, wound care, and care for chemotherapy or radiotherapy side effects. Treatment unmet needs included those related to discomfort associated with treatment, medical information asymmetry, and poor doctor–patient communication. Nutritional unmet needs included those related to nutrition information and nutritionist referrals. Psychosocial unmet needs included those related to emotional support, patients’ emotion problems, doubts toward treatments, and patients’ inability to accept their condition. Finally, economic unmet needs included those related to the financial burden on patients and their families.

### 2.3. Statistical Analysis

Descriptive statistics were first used to show distributions of participants’ demographic characteristics, NPC cancer stages, treatment phases and need domains. Proportional distributions of each need domain (yes or no) were calculated by 4 potentially associated factors: sex, age (<50, 50–59, and ≥60 years), cancer stage (I, II, III, and IV), and treatment phase (newly diagnosed, in-treatment, relapse, follow-up, and terminal care). We used Chi-square to examine the categorical data, or Fisher’s Exact test when numbers in the cells were small. T-test was used to compare mean ages. Patients <50 years old were considered as the younger and those ≥60 years old were as the older group. We further used Firth logistic regression analysis to estimate adjusted odds ratio (aOR) and 95% confidence interval (CI) of each unmet need domain by the 4 potentially associated factors [35,36]. IBM SPSS Statistics for Windows version 22.0 (IBM Corp., Armonk, NY, USA) was used to conduct statistical analysis. Statistical significance in this study was defined as *p* < 0.05.

## 3. Results 

### 3.1. Demographic Characteristics of NPC Patients

In total, 145 NPC patients were identified from consultations and services records, with more males than females (68.3% vs. 31.7%), and most cases aged less than 50 years (*n* = 87, 60.0%) with an average of 49.2 years and standard deviation (SD) of 10.3 years (Table 1). Patients with stage IV cancer constituted the largest group in the sample (*n* = 61, 42.1%). Only 16.5% of patients were diagnosed with clinical stages I and II. More than half of the participants met with the resource center team during the active treatment, while 20% had their consultation after they had been discharged from hospital. Among the unmet need domains, nutritional issue was the most prevalent unmet need (40.7%), followed by psychosocial problems (27.6%) and patient care (20.9%), and economic complaints the least (4.8%). 

### 3.2. Distribution of Patient Unmet Needs Across Different Domains

Table 2 shows that women had higher portions of unmet needs than men in health information and patient care, mainly reported when the cancer was diagnosed. The unmet needs on nutrition were similar in men and women. Older patients and patients with advanced stages were more likely to have nutritional unmet needs. Most of patients (75%, 44/59) reported nutritional unmet needs during the treatment period. Only 10.1% men reported unmet needs in treatment. 

### 3.3. Factors Associated with NPC Patients’ Unmet Needs

Table 3 shows that female NPC patients had an aOR of 2.47 (95% CI = 1.01–6.07) for the patient care need, compared with male patients. The older patients had an aOR of 9.39 (95% CI = 2.17–40.70) for the nutritional need, compared with younger patients. Compared with newly diagnosed patients, patients in the follow-up phase of treatment had lower levels of needs in health information (aOR = 0.10, 95% CI = 0.03–0.36), patient care (aOR = 0.35, 95% CI = 0.13–0.94), and nutrition (aOR = 0.18, 95% CI = 0.04–0.78). Psychosocial needs, however, were significantly higher (aOR = 4.67, 95% CI = 1.28–17.04) during the follow-up phase.

## 4. Discussion

The unmet supportive care needs in patients with cancer may vary by the type and stage of disease, healthcare availability, and patient characteristics. The present study indicated that unmet needs in NPC patients vary by demographic factors, cancer stage, and patient care phase. The unmet support care needs of these patients in this study were mostly noted at the newly diagnosed stage and in-treatment. New patients could be in a serious or life-threatening condition, suffering from a great anxiety of seeking medical care, when the cancer was diagnosed and announced. The majority of NPC patients are men in Taiwan. Over two third of our study population were men. Approximately 10% of male patients reported unmet need in treatment, whereas no female patients indicated this unmet need. Female patients in the present study had significantly greater needs in the patient care domain than did male patients. This may be because Asian women often serve as the main homemaker of the family. Therefore, even when such women are diagnosed with NPC, they may still need to deal with household chores and thus have an over two-fold greater desire of patient care. The female patients’ psychosocial need was also 1.98 times that of males, but not significant. A Korean study reported that 50.7% breast cancer patients had patient care needs [9]. A Malaysian breast cancer study found that the patient endorsement rate for the unmet patient care needs items in QoL of SCN-SF34 ranged from 29.9% to 45.3% [5]. 

When patients are diagnosed with cancer, they may lack of relevant knowledge about disease progression, examination procedures, treatment methods, treatment side effects, recovery, and follow-up care. Therefore, compared with patients in the follow-up stage, patients at newly diagnosed stage need more health information, patient care, and nutritional needs. Although medical technology in Taiwan is comparable to that of more developed societies, the reimbursement rates granted by Taiwan’s National Health Insurance to cover cancer diagnosis are low [4]; hence, a patient’s cancer diagnosis is directly delivered by the physician to the patient and the family after the physician examines the patient’s medical records. By contrast, higher diagnosis fees in Europe and the United States enable a series of procedures to be performed before the final delivery of a cancer diagnosis. First, an assessment of patient needs is performed. Then, the cancer diagnosis is delivered to the patient and their family by a multidisciplinary medical team comprising a physician psychologist, nurse, and social worker. This is followed by a detailed explanation of the treatment, patient care, nutritional, psychosocial, and economic support available to the patient. Additionally, the nurse–patient ratio in Taiwan (day shift: 1:8; evening shift: 1:13–1:15; night shift: 1:14–1:16) is higher than that in Europe and the United States. Therefore, nurses in Taiwan may be unable to provide sufficient explanation, information, and psychological support to patients newly diagnosed with cancer. A literature review on 94 studies investigating the unmet needs of patients with cancer concluded that most unmet needs occurred during the treatment period [6], while most health information needs occurred within one year after treatment [6,9]. The treatment and care mode of a patient may be relevant to their health insurance benefits.

The results of this study also indicate that female patients newly diagnosed with NPC have greater health information needs in addition to patient care domains. This may be because, before the commencement of treatment, patients may worry about the unresolved symptoms, pain, and anxiety related to the disease. Consequently, unmet health information and patient care needs can occur [37]. Young patients newly diagnosed with cancer may have unmet needs in the patient care domain. In addition to worrying about the disease, young patients with cancer worry about being unable to work and burdening their family with patient care duties [38]. The Korean study found that most breast cancer patients with shorter survival period had more unmet needs in relevant health information on pain management, wound care, and side effects from the treatment [9]. 

A Japanese study on women with advanced or recurrent breast cancer found that patients had strong psychosocial needs because of psychological distress and impaired QoL [39]. A cross-sectional study of 152 lung cancer patients in Taiwan found that symptoms of cancer and psychological distress substantially affect the unmet supportive care needs [40]. The top three domains of unmet needs they found were health information, patient care, and psychosocial needs. Patients in terminal care or relapse phase from our study did not report needs because only few patients in these two stages sought consultation service. 

Most patients with NPC live with impaired nasopharynx and pharyngeal recess. We observed that patient nutritional needs varied among age groups, cancer stages, and treatment phases, but not between men and women. Of 186 older adults, 36% were malnourished and 43% were at risk of malnutrition. The study also found that malnutrition in older adults led to weight-loss and loss of muscle mass [41]. Older patients exhibited much greater nutritional needs. A retrospective pooled analysis involving 4507 elderly patients of 12 countries found that malnutrition is often worsened by the degeneration of their digestive system, resulting reduction in nutrient absorption ability [31]. A hospital study in Taiwan found that most of NPC patients had Grade 2–3 xerostomia and mucositis [14]. An observational study on one NPC patient in Taiwan found that the patient suffered from swallowing difficulty because of mouth ulcerations, which may in turn lead to body weight loss [15]. Radiation therapy is likely to affect the nutrition intake in the prognosis leading to malnutrition and weight loss. Milko et al. found that a delicate food program can maintain good nutrition and offer an effective intervention to reduce malnutrition in older adults, thereby overall health and quality of life are improved [42].

About 28% of NPC patients in the present study had psychosocial needs mainly in the follow-up stage. It might be attributable to patients’ concerns about the future care and disease relapse after completing their treatment. They also tend to experience feelings of despair and have a fear of death. The anxiety can cause emotional instability, which consequently leads to poorer QoL and unmet psychosocial needs [5,10]. In contrast to the present study, an Australian study showed that patients with lung cancer in the treatment stage had greater psychosocial needs [11] Patients experienced fatigue and listlessness due to treatment side effects, leading to emotional instability and ultimately to psychosocial needs in patients. A systemic review on information needs in cancer survivors found that the provision of relevant health and patient care information is essential to eliminate anxiety and depression, and to improve their QoL [24]. The psychosocial needs of cancer patients might be related to their health information and patient care needs, and fulfilling these needs might in turn fulfill their psychosocial needs. A meta-analysis based on 12 studies evaluating the effectiveness of psychotherapy for incurable cancer patients concluded that the psychosocial needs of patients are mainly influenced by emotions of patients [43]. Appropriate psychological interventions could moderately reduce depression among patients. Very few participants reported unmet economic needs, which may be partly explained by cancer patients’ reduced healthcare co-payment obligations under the Taiwanese health insurance program.

This study has some limitations to consider. Secondary data were used; thus, the information extracted during data collection only pertained to what was available in the records identified from a single medical center in central Taiwan. No similar study has assessed the unmet needs for NPC patients at other medical centers in Taiwan. We are unable to evaluate whether our findings could be generalizable to all NPC patients in Taiwan, although distributions of sex and age in our study are similar to other studies. This study included approximately half of all NPC patients who attended the study center during the three-year study period. Individuals who chose not to attend the CRC or who attended the CRC but did not discuss their supportive care needs were not included. We were unable to assess how their exclusion may have potentially biased our findings. Information on patients’ supportive care needs was collected qualitatively through patient–clinician consultations and notes of these consultations were retrospectively coded against six supportive care need domains. The benefit of this approach was that the care team were able to identify areas outside of these six domains for which patients may have required support and referral. The limitation of this approach was that patients were not systematically asked about met and unmet need in each of the domains. As such, there is the potential that the proportion of participants who had unmet need may have been under-reported for some, or all, domains. This approach also meant that patients were not asked about specific issues to which their needs related or the degree to which these remained unmet and, as such, this study is unable to identify specific strategies that are likely to have the most impact on NPC patients’ supportive care. Future thematic analysis of the patient–clinician consultation notes could be performed to inform the development of a supportive care needs assessment tool for this specific patient population.

There is a need for future studies to investigate variation in unmet need between different areas of Taiwan, such as for NPC patients living in urban compared to non-urban areas. The development and validation of a needs assessment tool for this patient population could ensure that data collected from different groups are comparable. Most patients were interviewed during the inpatient treatment period and 40% of them had nutritional needs. No information on the oral treatment complications was documented. We are unable to confirm the nutrition need is due to impaired digest system. However, a food program that addresses the nutritional and biochemical needs of NPC patients should be developed [42].

## 5. Conclusions

Findings in this study suggest that unmet needs in patients with NPC cancer are associated with their sex, age, cancer stage, and treatment phase. Adequate health information and patient-centered care strategies are needed to provide to patients when the disease is newly diagnosed. The majority NPC patients were men who had higher treatment unmet need and economic unmet need, but the numbers of patients identified for both domains were small. Female patients may need attention because they have a greater unmet need in the patient care domain than male patients. Nutritional unmet need is most prevalent among the six domains of unmet needs and is particularly important for older patients. A nutritional care program should be developed. Medical staff should regularly assess the nutritional status of patients and give effective nutrition interventions to reduce the risk of malnutrition in NPC patients. Healthcare providers need to provide intervention to patients for reducing psychosocial needs, particularly in the follow-up stage. In short, appropriate and punctual support tailored provided by healthcare providers to address the unmet needs of patients would reduce the unmet supportive care needs for patients and also improve their quality of care. Future studies should develop a systematic tool to assess both unmet and met needs in all patients. A larger sample size is required. Ultimately, the overall QoL of patients can be improved.

## Figures and Tables

**Table 1:** Distribution of demographic and disease characteristics of NPC patients and Unmet need reported in six domains.

Variable	*N*	%
Total	145	100.0
Sex		
Male	99	68.3
Female	46	31.7
Age (year) (Mean ± SD)	49.2 ± 10.3	
<50	87	60.0
50–59	40	27.6
≥60	18	12.4
Cancer stage		
I	7	4.8
II	17	11.7
III	60	41.4
IV	61	42.1
Treatment phase		
Newly diagnosed	26	17.9
In-treatment	85	58.6
Relapse	2	1.4
Follow up	29	20.0
Terminal care	3	2.1
Unmet need in		
Health information		
Yes	14	9.7
No	131	90.3
Patient care		
Yes	30	20.9
No	115	79.1
Treatment		
Yes	10	6.9
No	135	93.1
Nutritional		
Yes	59	40.7
No	86	59.3
Psychosocial		
Yes	40	27.6
No	105	72.4
Economic		
Yes	7	4.8
No	138	95.2

**Table 2:** Distributions of NPC patient needs across the six domains by sex, age, cancer stage and treatment phase.

Variable	Health Information			Patient Care			Treatment			Nutritional			Psychosocial			Economic		
	No	Yes	*p*	No	Yes	*p*	No	Yes	*p*	No	Yes	*p*	No	Yes	*p*	No	Yes	*p*
	*n* (%)	*n* (%)		*n* (%)	*n* (%)		*n* (%)	*n* (%)		*n* (%)	*n* (%)		*n* (%)	*n* (%)		*n* (%)	*n* (%)	
Total	131 (90.3)	14 (9.7)		115 (79.1)	30 (20.9)		135 (93.1)	10 (6.9)		86 (59.3)	59 (40.7)		105 (72.4)	40 (27.6)		138 (95.2)	7 (4.8)	
Sex ^1^			<0.001			0.026			<0.001			0.148			0.489			<0.001
Male	91 (91.9)	8 (8.1)		84 (84.8)	15 (15.2)		89 (89.9)	10 (10.1)		58 (58.6)	41 (41.4)		76 (76.8)	23 (23.2)		93 (93.9)	6 (6.1)	
Female	40 (87.0)	6 (13.0)		31 (67.4)	15 (32.6)		46 (100.0)	-		28 (60.9)	18 (39.1)		29 (63.0)	17 (37.0)		45 (97.8)	1 (2.2)	
Age (year)																		
Mean ± SD ^2^	49.5 ± 10.5	46.9 ± 8.6	0.348	49.27 ± 9.8	49.07 ± 12.2	0.792	49.0 ± 10.6	52.0 ± 4.5	0.260	47.3 ± 9.9	52.0 ± 10.5	0.023	50.9 ± 9.7	45.0 ± 10.8	0.018	49.4 ± 10.5	45.6 ± 3.8	0.153
<50	79 (90.8)	8 (9.2)	0.222	68 (78.2)	19 (21.8)	0.480	84 (96.6)	3 (3.4)	0.011	56 (64.4)	31 (35.6)	<0.001	57 (65.5)	30 (34.5)	0.004	80 (92.0)	7 (8.0)	0.138
50–59	34 (85.0)	6 (15.0)		34 (85.0)	6 (15.0)		33 (82.5)	7 (17.5)		27 (67.5)	13 (32.5)		30 (75.0)	10 (25.0)		40 (100.0)	-	
≥60	18 (100.0)	-		13 (72.2)	5 (27.8)		18 (100.0)	-		3 (16.7)	15 (83.3)		18 (100.0)	-		18 (100.0)	-	
Cancer stage			0.097			0.855			0.653			0.040			0.897			0.496
I	6 (85.7)	1 (14.3)		5 (71.4)	2 (28.6)		7 (100.0)	-		4 (57.1)	3 (42.9)		6 (85.7)	1 (14.3)		6 (85.7)	1 (14.3)	
II	13 (76.5)	4 (23.5)		13 (76.5)	4 (23.5)		17 (100.0)	-		7 (41.2)	10 (58.8)		13 (76.5)	4 (23.5)		17 (100.0)	-	
III	54 (90.0)	6 (10.0)		49 (81.7)	11 (18.3)		56 (93.3)	4 (6.7)		31 (51.7)	29 (48.3)		42 (70.0)	18 (30.0)		57 (95.0)	3 (5.0)	
IV	58 (95.1)	3 (4.9)		48 (78.7)	13 (21.3)		55 (90.2)	6 (9.8)		44 (72.1)	17 (27.9)		44 (72.1)	17 (27.9)		58(95.1)	3 (4.9)	
Treatment phases			<0.001			0.021			0.295			<0.001			0.006			0.008
Newly diagnosed	16 (61.5)	10 (38.5)		15 (57.7)	11 (42.3)		25 (96.2)	1 (3.8)		15 (57.7)	11 (42.3)		20 (76.9)	6 (23.1)		26 (100.0)	-	
In-treatment	82 (96.5)	3 (3.5)		68 (80.0)	17 (20.0)		79 (92.9)	6 (7.1)		41 (48.2)	44 (51.8)		67 (78.8)	18 (21.2)		80 (94.1)	5 (5.9)	
Relapse	2 (100.0)	-		2 (100.0)	-		1 (50.0)	1 (50.0)		1 (50.0)	1 (50.0)		2 (100.0)	-		2 (100.0)	-	
Follow up	28 (96.6)	1 (3.4)		27 (93.1)	2 (6.9)		27 (93.1)	2 (6.9)		26 (89.7)	3 (10.3)		13 (44.8)	16 (55.2)		29 (100.0)	-	
Terminal care	3 (100.0)	-		3 (100.0)	-		3 (100.0)	-		3 (100.0)	-		3 (100.0)	-		1 (33.3)	2 (66.7)	

^1^ Used Chi-square test or Fisher’s exact test for categorical data. ^2^ Used *t*-test.

**Table 3:** Firth logistic regression estimated odds ratio of unmet needs associated with sex, age, cancer stage, and treatment phase.

Variable	Unmet Needs ^1^							
	Health Information		Patient Care		Nutritional		Psychosocial	
	Adjusted OR ^2^	95% CI ^2,3^	Adjusted OR ^2^	95% CI ^2,3^	Adjusted OR ^2^	95% CI ^2,3^	Adjusted OR ^2^	95% CI ^2,3^
Sex								
Male (Ref.)	1		1		1		1	
Female	1.57	0.40–6.21	2.47	1.01–6.07 *	0.72	0.30–1.74	1.95	0.80–4.75
Age (year)								
<50 (Ref.)	1		1		1		1	
50–59	2.70	0.59–12.90	1.05	0.36–3.06	0.81	0.33-2.01	0.62	0.24–1.61
≥60	-	-	1.71	0.47–6.15	9.39	2.17–40.70 **	-	-
Cancer stage								
I (Ref.)	1		1		1		1	
II	1.13	0.09–14.90	0.71	0.09–5.57	2.48	0.32–19.00	1.11	0.11–11.40
III	0.61	0.06–6.19	0.96	0.15–6.24	2.81	0.46–17.10	0.95	0.12–7.56
IV	0.50	0.05–5.56	1.23	0.19–7.84	0.98	0.16–6.03	0.82	0.10–6.75
Treatment phases								
Newly diagnosed (Ref.)	1		1		1		1	
In-treatment	0.10	0.03–0.36 ***	0.35	0.13–0.94 *	1.41	0.54–3.69	1.14	0.37–3.51
Relapse	-	-	-	-	0.65	0.01–31.10	-	-
Follow up	0.09	0.02–0.61 *	0.12	0.03–0.58 **	0.18	0.04–0.78 *	4.67	1.28–17.04 *
Terminal care	-	-	-	-	-	-	-	-

^1^ The treatment and economic domains were dropped from this analysis due to its zero count and small sizes in cells. We were unable to calculate odds ratio for cells with zero and marked with hyphen. ^2^ Abbreviations: OR, odds ratio; CI, confidence interval. ^3^ * *p*-value < 0.05, ** *p*-value < 0.01, *** *p*-value < 0.001.

## References

[1] Bray F., Ferlay J., Soerjomataram I., Siegel R.L., Torre L.A., Jemal A. (2018). Global cancer statistics 2018: GLOBOCAN estimates of incidence and mortality worldwide for 36 cancers in 185 countries. CA Cancer J. Clin..

[2] Jin J., Ouyang Z., Wang Z. (2014). Association of fruit and vegetables with the risk of nasopharyngeal cancer: Evidence from a meta-analysis. Sci. Rep..

[3] Yong S.K., Ha T.C., Yeo M.C., Gaborieau V., McKay J.D., Wee J. (2017). Associations of lifestyle and diet with the risk of nasopharyngeal carcinoma in Singapore: A case-control study. Chin. J. Cancer.

[4] Health Promotion Administration, Cancer Registry Annual Report 2012–2016 Ministry of Health and Welfare, Taiwan. https://www.hpa.gov.tw/Pages/List.aspx?nodeid=269..

[5] Edib Z., Kumarasamy V., Binti Abdullah N., Rizal A.M., Al-Dubai S.A. (2016). Most prevalent unmet supportive care needs and quality of life of breast cancer patients in a tertiary hospital in Malaysia. Health Qual. Life Outcomes.

[6] Harrison J.D., Young J.M., Price M.A., Butow P.N., Solomon M.J. (2009). What are the unmet supportive care needs of people with cancer? A systematic review. Support. Care Cancer.

[7] Langius J.A., Bakker S., Rietveld D.H., Kruizenga H.M., Langendijk J.A., Weijs P.J., Leemans C.R. (2013). Critical weight loss is a major prognostic indicator for disease-specific survival in patients with head and neck cancer receiving radiotherapy. Br. J. Cancer.

[8] Li W.W., Lam W.W., Au A.H., Ye M., Law W.L., Poon J., Kwong A., Suen D., Tsang J., Girgis A. (2013). Interpreting differences in patterns of supportive care needs between patients with breast cancer and patients with colorectal cancer. Psychooncology.

[9] Park B.W., Hwang S.Y. (2012). Unmet needs of breast cancer patients relative to survival duration. Yonsei Med. J..

[10] So W.K., Choi K.C., Chen J.M., Chan C.W., Chair S.Y., Fung O.W., Wan R.W., Mak S.S., Ling W.M., Ng W.T. (2014). Quality of life in head and neck cancer survivors at 1 year after treatment: The mediating role of unmet supportive care needs. Support. Care Cancer.

[11] Ugalde A., Aranda S., Krishnasamy M., Ball D., Schofield P. (2012). Unmet needs and distress in people with inoperable lung cancer at the commencement of treatment. Support. Care Cancer.

[12] Lu C.S., Peng Y.J., Kao H.W., Chen H.C., Ho C.L., Chang P.Y. (2015). Nasopharyngeal Myoepithelial Carcinoma Mimicking Nasopharyngeal Carcinoma. J. Cancer Res. Pract..

[13] Xiao W.W., Huang S.M., Han F., Wu S.X., Lu L.X., Lin C.G., Deng X.W., Lu T.X., Cui N.J., Zhao C. (2011). Local control, survival, and late toxicities of locally advanced nasopharyngeal carcinoma treated by simultaneous modulated accelerated radiotherapy combined with cisplatin concurrent chemotherapy: Long-term results of a phase 2 study. Cancer.

[14] Kong F., Ying H., Huang S., Du C., Zhou J., Hu C. (2014). Preliminary results of nasopharyngeal carcinoma treated with intensity-modulated radiotherapy: A retrospective study of 364 patients. Eur. Arch. Otorhinolaryngol..

[15] Sucipto U., Waluyo A., Yona S. (2019). Phenomenological study: The experiences of patients with nasopharyngeal cancer after undergoing chemoradiation. BMC Nurs..

[16] Vangelov B., Venchiarutti R.L., Smee R.I. (2017). Critical Weight Loss in Patients with Oropharynx Cancer During Radiotherapy (+/- Chemotherapy). Nutr. Cancer.

[17] Favaro-Moreira N.C., Krausch-Hofmann S., Matthys C., Vereecken C., Vanhauwaert E., Declercq A., Bekkering G.E., Duyck J. (2016). Risk Factors for Malnutrition in Older Adults: A Systematic Review of the Literature Based on Longitudinal Data. Adv. Nutr..

[18] Rosedale M., Fu M.R. (2010). Confronting the unexpected: Temporal, situational, and attributive dimensions of distressing symptom experience for breast cancer survivors. Oncol. Nurs. Forum.

[19] Shi Q., Smith T.G., Michonski J.D., Stein K.D., Kaw C., Cleeland C.S. (2011). Symptom burden in cancer survivors 1 year after diagnosis: A report from the American Cancer Society’s Studies of Cancer Survivors. Cancer.

[20] Lu X., Hui-Chan C.W., Tsang W.W. (2013). Effects of Tai Chi training on arterial compliance and muscle strength in female seniors: A randomized clinical trial. Eur. J. Prev. Cardiol..

[21] Liao M.N., Chen S.C., Lin Y.C., Chen M.F., Wang C.H., Jane S.W. (2014). Education and psychological support meet the supportive care needs of Taiwanese women three months after surgery for newly diagnosed breast cancer: A non-randomised quasi-experimental study. Int. J. Nurs. Stud..

[22] Wang T., Molassiotis A., Chung B.P.M., Tan J.Y. (2018). Unmet care needs of advanced cancer patients and their informal caregivers: A systematic review. BMC Palliat. Care.

[23] Boyes A.W., Girgis A., D’Este C., Zucca A.C. (2012). Prevalence and correlates of cancer survivors’ supportive care needs 6 months after diagnosis: A population-based cross-sectional study. BMC Cancer.

[24] Husson O., Mols F., van de Poll-Franse L.V. (2011). The relation between information provision and health-related quality of life, anxiety and depression among cancer survivors: A systematic review. Ann. Oncol..

[25] Usta Y.Y. (2012). Importance of social support in cancer patients. Asian Pac. J. Cancer Prev..

[26] Bjorneklett H.G., Rosenblad A., Lindemalm C., Ojutkangas M.L., Letocha H., Strang P., Bergkvist L. (2013). Long-term follow-up of a randomized study of support group intervention in women with primary breast cancer. J. Psychosom. Res..

[27] Barg F.K., Cronholm P.F., Straton J.B., Keddem S., Knott K., Grater J., Houts P., Palmer S.C. (2007). Unmet psychosocial needs of Pennsylvanians with cancer: 1986–2005. Cancer.

[28] Fang F.M., Tsai W.L., Lee T.F., Liao K.C., Chen H.C., Hsu H.C. (2010). Multivariate analysis of quality of life outcome for nasopharyngeal carcinoma patients after treatment. Radiother. Oncol..

[29] Abdullah N.A., Wan Mahiyuddin W.R., Muhammad N.A., Ali Z.M., Ibrahim L., Ibrahim Tamim N.S., Mustafa A.N., Kamaluddin M.A. (2013). Survival rate of breast cancer patients in Malaysia: A population-based study. Asian Pac. J. Cancer Prev..

[30] Lis C.G., Gupta D., Lammersfeld C.A., Markman M., Vashi P.G. (2012). Role of nutritional status in predicting quality of life outcomes in cancer—A systematic review of the epidemiological literature. Nutr. J..

[31] Kaiser M.J., Bauer J.M., Ramsch C., Uter W., Guigoz Y., Cederholm T., Thomas D.R., Anthony P.S., Charlton K.E., Maggio M. (2010). Frequency of malnutrition in older adults: A multinational perspective using the mini nutritional assessment. J. Am. Geriatr. Soc..

[32] Qiu C., Yang N., Tian G., Liu H. (2011). Weight loss during radiotherapy for nasopharyngeal carcinoma: A prospective study from northern China. Nutr. Cancer.

[33] Lee H.O., Lee J.J. (2015). Nutritional intervention using nutrition care process in a malnourished patient with chemotherapy side effects. Clin. Nutr. Res..

[34] Huang T.L., Chien C.Y., Tsai W.L., Liao K.C., Chou S.Y., Lin H.C., Dean Luo S., Lee T.F., Lee C.H., Fang F.M. (2016). Long-term late toxicities and quality of life for survivors of nasopharyngeal carcinoma treated with intensity-modulated radiotherapy versus non-intensity-modulated radiotherapy. Head Neck.

[35] Wang X. (2014). Firth logistic regression for rare variant association tests. Front. Genet..

[36] Puhr R., Heinze G., Nold M., Lusa L., Geroldinger A. (2017). Firth’s logistic regression with rare events: Accurate effect estimates and predictions?. Stat. Med..

[37] Chen S.C., Lai Y.H., Liao C.T., Chang J.T., Lin C.Y., Fan K.H., Huang B.S. (2013). Supportive care needs in newly diagnosed oral cavity cancer patients receiving radiation therapy. Psychooncology.

[38] Zebrack B.J., Block R., Hayes-Lattin B., Embry L., Aguilar C., Meeske K.A., Li Y., Butler M., Cole S. (2013). Psychosocial service use and unmet need among recently diagnosed adolescent and young adult cancer patients. Cancer.

[39] Uchida M., Akechi T., Okuyama T., Sagawa R., Nakaguchi T., Endo C., Yamashita H., Toyama T., Furukawa T.A. (2011). Patients’ supportive care needs and psychological distress in advanced breast cancer patients in Japan. Jpn. J. Clin. Oncol..

[40] Liao Y.C., Liao W.Y., Shun S.C., Yu C.J., Yang P.C., Lai Y.H. (2011). Symptoms, psychological distress, and supportive care needs in lung cancer patients. Support. Care Cancer.

[41] Papparotto C., Bidoli E., Palese A. (2013). Risk factors associated with malnutrition in older adults living in Italian nursing homes: A cross-sectional study. Res. Gerontol. Nurs..

[42] Zanini M., Bagnasco A., Catania G., Aleo G., Sartini M., Cristina M.L., Ripamonti S., Monacelli F., Odetti P., Sasso L. (2017). A Dedicated Nutritional Care Program (NUTRICARE) to reduce malnutrition in institutionalised dysphagic older people: A quasi-experimental study. J. Clin. Nurs..

[43] Okuyama T., Akechi T., Mackenzie L., Furukawa T.A. (2017). Psychotherapy for depression among advanced, incurable cancer patients: A systematic review and meta-analysis. Cancer Treat. Rev..

